# Effect of intranasal administration of concentrated growth factors on regeneration of the olfactory epithelium in an olfactory dysfunction-induced rat model

**DOI:** 10.1371/journal.pone.0298640

**Published:** 2024-02-28

**Authors:** Naruhiko Kai, Naoya Nishida, Kunihide Aoishi, Taro Takagi, Naohito Hato

**Affiliations:** 1 Department of Otolaryngology, Head and Neck Surgery, Ehime University Graduate School of Medicine, Shitsukawa, Toon, Ehime, Japan; 2 Department of Otolaryngology, Ehime Prefectural Niihama Hospital, Niihama, Japan; Monell Chemical Senses Center, UNITED STATES

## Abstract

**Objective:**

The development of treatments that promote the regenerative capacity of the olfactory epithelium (OE) is desirable. This study aimed to evaluate the effects of intranasal administration of concentrated growth factors (CGFs) in a rat model of olfactory dysfunction.

**Study design:**

Animal study.

**Methods:**

Nineteen male rats were used. Fourteen olfactory dysfunction models were created by intraperitoneal administration of 3-methylindole. We randomly divided the rats from the olfactory dysfunction model after 1 week into the CGF or saline group; CGFs were administered to seven animals and saline to seven animals. Behavioral assessments using the avoidance test were conducted until day 28 after CGF/saline administration. On day 28, histological evaluation was conducted to determine olfactory epithelial thickness and the olfactory marker protein (OMP)-positive cell count. Five animals were intraperitoneally injected with saline as the control group.

**Results:**

The avoidance rate remained decreased until 28 days after CGF/saline administration, and there was no significant difference between the two groups. Olfactory epithelial thicknesses on day 28 were 38.64 ± 3.17 μm and 32.84 ± 4.50 μm in the CGF and saline groups, respectively. OE thickness was significantly thicker in the CGF group than in the saline group (*P* = 0.013). The numbers of OMP-positive cells were 40.29 ± 9.77/1.0 × 10^4^ μm^2^ and 31.00 ± 3.69/1.0 × 10^4^ μm^2^ in the CGF and saline groups, respectively. The number of OMP+ cells in the CGF group was significantly increased compared with that in the saline group (*P* = 0.009). Both groups showed no improvement compared with the control group (OE thickness: 54.08 ± 3.36 μm; OMP+ cell count: 56.90 ± 9.91/1.0 × 104 μm^2^).

**Conclusions:**

The CGF group showed improved olfactory epithelial thickness and OMP-positive cell numbers compared with that in the saline group.

## Introduction

Patients with olfactory dysfunction have a decreased quality of life and are exposed to daily hazards such as food spoilage, gas leaks, and smoke inhalation [1]. The prevalence of olfactory dysfunction in the United States is approximately 1–3% of the population [2, 3]. Sensorineural dysfunction (e.g., degeneration of the olfactory epithelium [OE] and nerves caused by viral infection and drug-induced dysfunction) leads to olfactory epithelial damage caused by degeneration and shedding of the OE, which correlates with the degree of olfactory dysfunction [4, 5]. The efficacy of various drugs, such as zinc preparations and vitamin A, has been reported for sensorineural dysfunction in clinical practice; however, there are no data showing significant differences in blind placebo-controlled studies [6, 7].

In the OE, olfactory receptor neurons (ORNs) have a unique regenerative capacity to undergo continuous neurogenesis under physiological and damage conditions [8]. Regenerated ORNs extend their axons into the olfactory bulb (OB) and reassemble synapses with the mitral cells in the OB, thereby regenerating the olfactory conduction tract [9]. Animal models have shown that olfactory neuron regeneration is induced by neurotrophins, such as nerve growth factor (NGF) [10] and brain-derived neurotrophic factor (BDNF) [11], and growth factors such as basic fibroblast growth factor (bFGF) [12], insulin-like growth factor (IGF) [13], and transforming growth factor-α (TDF-α) [14]. The promotion of regeneration in the OE by these growth factors is expected to lead to the treatment of olfactory dysfunction [15]. Owing to the global outbreak of coronavirus disease (COVID-19) in 2019, the incidence of olfactory dysfunction is increasing, so it is desirable to develop treatments that promote the regenerative capacity of the OE.

Recently, Yasak et al. showed that platelet-rich plasma (PRP) promotes olfactory epithelial regeneration by administering PRP to olfactory dysfunction models [16]. PRP is a platelet concentration obtained by centrifuging blood, and it contains many growth and neurotrophic factors that have been clinically applied to promote and accelerate healing [17]. Several clinical treatment studies have reported that topical injections of PRP for olfactory dysfunction after COVID-19 improved TDI-score and visual analog scale scores [18–20]. However, PRP administration requires topical injection into the olfactory cleft, which may cause pain and bleeding [19].

Platelet-rich fibrin (PRF) and concentrated growth factors (CGFs) are also autologous platelet concentrates. RPF and CGFs are prepared with simpler protocols than PRP and do not require the addition of anticoagulants, thrombin, or calcium chloride [21]. Kobayashi et al. demonstrated that PRF is more potent in angiogenesis than PRP [22]. Studies have reported that CGFs and PRF have similar mechanical properties, degradability, and major growth factor content; both superior to PRP [23, 24]. In addition, PRF and CGFs have the ability to stimulate continuous and stable release of all growth factors over 14 days [25]. Due to differences in centrifugation rates, CGFs separates as a larger, denser, growth factor-rich fibrin matrix than PRF [26]. Lee et al. reported that the tensile strength and growth factor content of CGFs were significantly higher compared to that of PRF [27]. Li et al. reported that CGFs showed more effective long-term osteoinductive and tissue regenerative potential than PRP or PRF [28].

Therefore, we focused on CGFs. The administration of CGFs into the olfactory cleft, which does not require topical injection such as the administration of PRP, is considered more suitable than PRP administration as a technique that provides growth factors to the OE. We hypothesized that intranasal administration of CGFs could be an ideal treatment for patients with olfactory dysfunction. This study aimed to investigate the effects of CGFs on the OE in a rat model with olfactory dysfunction.

## Materials and methods

### Ethics statements

The study protocol was approved by the Ethics Committee for Animal Experiments of Ehime University (number: 05HI86-2). All methods were performed in accordance with relevant guidelines and regulations. This study was conducted in accordance with ARRIVE guidelines (https://arriveguidelines.org).

### Animal preparation and 3-methylindole injection

We used 12-week-old male Wister/ST rats (n = 19) purchased from Japan SLC, Inc. (Shizuoka, Japan). All rats were grown in acrylic cages with woodchip bedding at a temperature of 22 ± 1°C with a 12-hour cycle. The rats had free access to food. To evaluate behavior based on water intake, plain water and water scented with vanillin were prepared. The water was placed in a bottle and replaced weekly. Olfactory dysfunction was induced in all rats by an intraperitoneal injection of 3-methylindole (3-MI; #228–00685; FUJIFILM Wako Pure Chemical Corporation, Tokyo, Japan) dissolved in corn oil, and administered at 300 mg/kg [29, 30]. Rats (n = 5) intraperitoneally injected with saline were used as controls to observe healthy olfactory epithelial tissues.

### Avoidance test

A two-bottle preference test using vanillin was conducted as a behavioral test to evaluate olfactory function [31]. The 0.1% vanillin solution did not induce a gustatory response and the rodents tended to dislike the smell of vanillin. Therefore, an increase in the consumption rate of 0.1% aqueous vanillin solution would imply a decrease in olfactory function [32]. Bottles containing only distilled water and bottles containing vanillin-water (0.1% vanillin [FUJIFILM Wako Pure Chemical Corporation] dissolved in distilled water) were placed in the cages, and bottle positions were changed daily. Bottle content intake was measured every other week, and avoidance was calculated by dividing normal water intake by the sum of normal water intake and vanillin-water intake. An avoidance ratio close to 100% indicates healthy olfactory function, whereas an avoidance ratio close to 50% indicates that the animals drank the two waters equally and were unable to distinguish the smell of vanillin. In other words, the rats were in a state of olfactory dysfunction.

### Preparation and intranasal administration of CGFs

Blood samples (8–10 cc) were taken from healthy rats. The blood was quickly transferred to a vacuum tube and centrifuged on the CGF setting of a Medifuge centrifugation system (Silfradent S. R. L., Santa Sofia, Italy) for approximately 13 minutes. The gel-like material in the middle layer of the trilayer structure was used as CGFs [26]. CGFs were administered intranasally 1 week later after 3-MI intraperitoneally (i.p.). Rats were anesthetized with medetomidine (0.15 mg/kg), midazolam (2 mg/kg), and butorphanol (2.5 mg/kg). CGFs were subdivided, and 50 μL of the CGFs was administered into both nasal cavities with a micropipette. Rats were sedated in the supine position for 30 minutes after administration to ensure CGF infiltration into the olfactory epithelial distribution area. This procedure was repeated across consecutive days. The first day of CGF administration was defined as day 0 and the CGF was administered on days 0, 1, 2. In the control group, saline was administered to the nasal cavity using the same procedure. The group administrated CGFs was defined the CGF group (n = 7), whereas the group administrated saline was defined the saline group (n = 7).

### Nasal tissue preparation

Under deep anesthesia with isoflurane, all rats were sacrificed on day 28, and a 4% paraformaldehyde fixative was perfused from the left ventricle. Heads were fixed with 10% formaldehyde at a temperature of 25 ± 1°C for 24 hours. The specimens were decalcified in a decalcification solution (10% ethylenediamine tetraacetic acid, pH 7.0) for 7 days, dehydrated using a graded alcohol and xylene series, and embedded in paraffin.

### Histological and immunohistochemical examination

Whole-head samples were cut in coronal sections at the level of the anterior edge of the olfactory bulb, which were serially sectioned to 4.5-μm thickness. The sections were deparaffinized and rehydrated using a graded xylene and alcohol series. One section was stained with hematoxylin and eosin and used for thickness measurements, and the other was used for immunohistochemistry with olfactory marker protein (OMP) expressed in mature ORNs. The sections were placed in 0.01 M of citric acid buffer solution and heated at 100°C in a microwave oven for 20 minutes for antigen retrieval.

Endogenous peroxidase was blocked with 3% hydrogen peroxide, and nonspecific binding was blocked with an R.T.U. Animal Free Blocker and Diluent (SP-5035, Vector Laboratories, Burlingame, CA, USA). After rinsing with phosphate buffer saline (PBS), the sections were incubated with goat polyclonal antibodies against OMP (1:900; #019–22291;FUJIFILM Wako Pure Chemical Corporation) overnight at 4°C. After rinsing with PBS, the sections were incubated with a secondary antibody (Histofine Simple Stain Mouse MAX-PO(R) #414,341; Nichirei Biosciences, Tokyo, Japan) for 1 hour at room temperature and stained with DAB chromogen for 2 minutes. After a final rinse, counterstaining was performed using the Mayer hematoxylin stain. Three sections were randomly selected from the sections prepared, and one location was used as the measurement point in each section. Measurements were taken on the septum 1–3 mm below the upper end of the nasal cavity from either the right or left side of the nasal cavity. The number of OMP-positive cells was counted per 1.0 × 10^4^ μm^2 ^of OE. Measurements from three sections per animal are averaged to yield a single value per animal, and are then statistically analyzed.

### Statistical analysis

All statistical analyses were performed using EZR (Saitama Medical Center, Jichi Medical University, Saitama, Japan), which is a graphical user interface for R (The R Foundation for Statistical Computing, Vienna, Austria). More precisely, it is a modified version of the R commander designed to add statistical functions frequently used in biostatistics [33]. Comparisons between two independent groups were made using the Mann–Whitney U test. The significance level was set at P < 0.05.

## Results

### Avoidance test

Table 1 shows the avoidance rates before 3-MI administration and on days 0, 7, 14, 21, and 28. Both the CGF and saline groups showed a more than 80% avoidance rate before 3-MI administration. The avoidance rates of both groups decreased to 64.70 ± 0.13% and 58.1 ± 0.16%, respectively, on day 0. The avoidance rates of both groups remained decreased until day 28, and no significant differences were observed between the two groups during any of these time periods.

**Table 1:** Avoidance rate from before intraperitoneal 3-MI administration to day 28 in the saline and CGF groups.

	Saline	CGF	*P*-value
	Average (%) ± SD	Average (%) ± SD	
**Pre-3-MI**	81.56 ± 0.07	80.28 ± 0.07	0.902
**Day 0**	64.70 ± 0.13	58.17 ± 0.16	0.456
**Day 7**	56.91 ± 0.07	60.22 ± 0.10	0.710
**Day 14**	62.80 ± 0.10	57.11 ± 0.11	0.482
**Day 21**	63.60 ± 0.08	59.03 ± 0.12	0.620
**Day 28**	62.53 ± 0.17	57.83 ± 0.14	0.805

CGF, concentrated growth factor; SD, standard deviation; 3-MI, 3-methylindole

### Olfactory epithelial thickness and OMP immunohistochemistry in the OE

**Fig 1:**
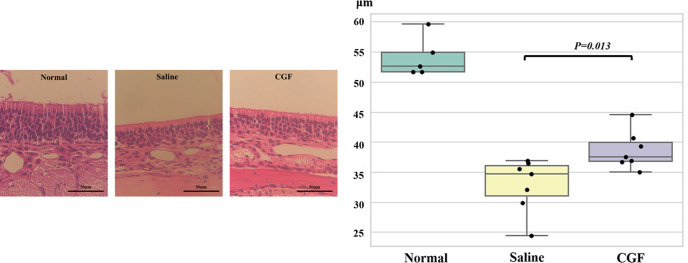
Comparison of the olfactory epithelial thickness in the saline and CGF groups. Left: Representative images of hematoxylin-eosin staining of the olfactory epithelium in the control, saline, and CGF groups on day 28 are shown. Right: The olfactory epithelial thickness is thicker in the CGF group than in the saline group (P = 0.013). However, it is not improved compared with the thickness of the control group. CGF, concentrated growth factor.

Fig 1 shows the tissue images of the hematoxylin and eosin-stained OE on day 28 and a comparison of the thickness of the OE in each group. The olfactory epithelial thicknesses were 38.64 ± 3.17 μm and 32.84 ± 4.50 μm in the CGF and saline groups, respectively; the olfactory epithelial thickness was significantly thicker in the CGF group than in the saline group (*P* = 0.013).

However, neither group showed an improvement in the thickness compared with the control group (54.08 ± 3.36 μm).

**Fig 2:**
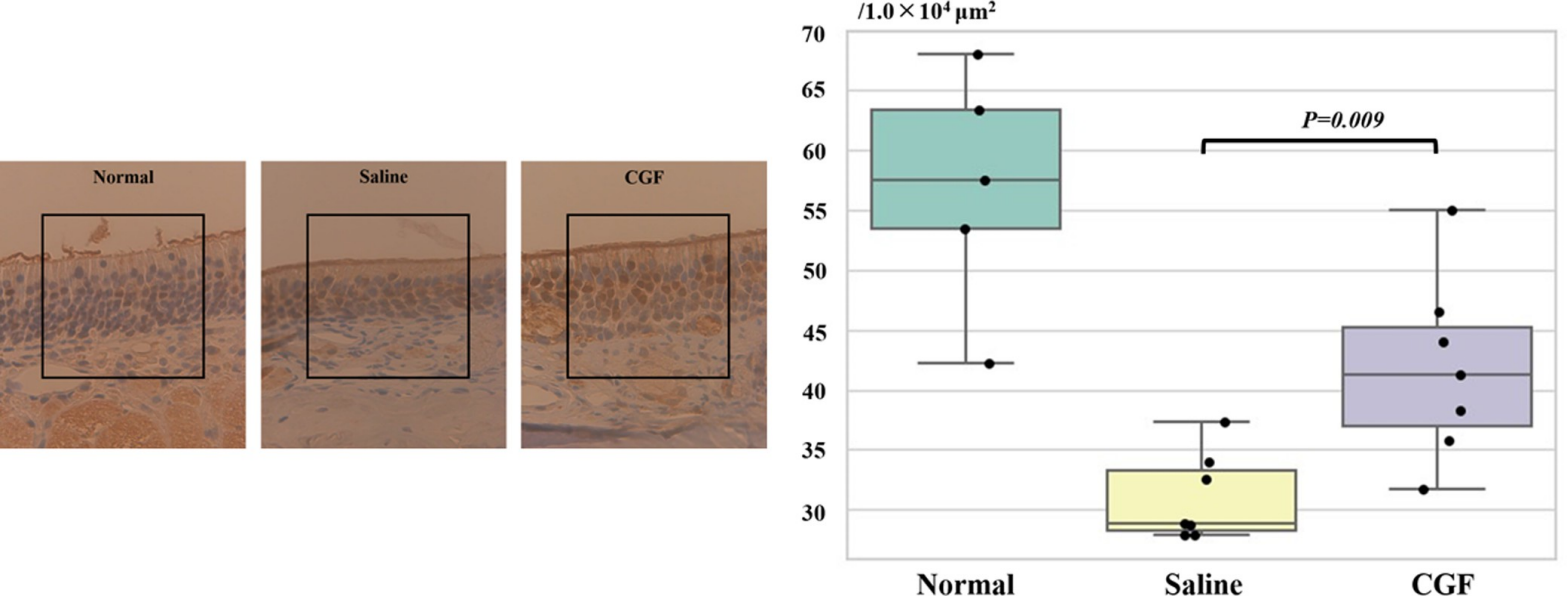
Comparison of the number of OMP-positive cells in the saline and CGF groups. Left: Representative images of OMP immunostaining of the OE of the healthy, saline, and CGF groups on day 28 are shown. The square scale in the figure is 100 μm × 100 μm. The number of OMP-positive cells in this square scale was counted. Right: The number of OMP-positive cells of the CGF group is higher in the CGF group than in the saline group (*P* = 0.009). However, the value did not improve to the number of OMP-positive cells in the healthy group. CGF, concentrated growth factor; OE, olfactory epithelium; OMP, olfactory marker protein.

Fig 2 shows tissue images of OMP immunostaining of the OE on day 28 and a comparison of the number of OMP-positive cells in each group. The numbers of OMP-positive cells were 40.29 ± 9.77/1.0 × 10^4^ μm^2^ and 31.00 ± 3.69/1.0 × 10^4^ μm^2^ in the CGF and saline groups, respectively; the number of OMP-positive cells was significantly higher in the CGF group than in the saline group (*P* = 0.009). However, the number of OMP-positive cells in both groups was significantly lower when compared with that in the healthy group (56.90 ± 9.91/1.0 × 10^4^ μm^2^).

## Discussion

We investigated the effects of CGFs on OE in an olfactory dysfunction rat model. In clinical practice, there are a small number of patients with persistent olfactory dysfunction who do not improve after treatment [34]. In this experiment, we used 3-MI, which is strongly impairing; because we envisioned an approach for patients with long-lasting olfactory dysfunction. 3-MI was chosen as a nasal toxicant because its effect appears to be secondary to bioactivation of P450 enzymes in supporting cells, resulting in the production of free radicals [35]. Methimazole is also used to induce olfactory dysfunction, but it has been reported that olfactory epithelial damage caused by methimazole normalizes after approximately 4 weeks [31]. 3-MI causes dose-dependent degeneration of the OE and severe olfactory dysfunction at doses of 300 and 400 mg/kg [36]. Li et al. showed a histological decrease in OE to about 1/4 of its thickness on day 3 after 3-MI administration, and only 1/2 of its thickness had improved by 28 days. In addition, they showed that the persistent impairment of olfactory behavior lasted 4 weeks [37]. Almost all mature ORNs drop out within 1 week after 3-MI administration, and olfactory dysfunction persists [30]. This study showed that 3-MI-induced olfactory epithelial damage was not normal, even after 4 weeks. Even in this strongly damaged model, the CGF group showed improved olfactory epithelial thickness and OMP-positive cell numbers compared with the saline group.

CGFs contain several growth factors including bFGF and IGF-1 that are released over time [38]. The bFGF receptor has been identified in the OE [39], and IGF-1 receptor mRNA expression is increased early during olfactory epithelial injury, suggesting that IGF-1 contributes to olfactory epithelial regeneration [13]. In addition, the target cells of CGF are considered to be stem cells [40, 41]. It has been reported that olfactory epithelial basal cells are stem cells because olfactory epithelial basal cells expresses matrix metalloproteinase-2 (MMP-2) to immature olfactory neurons within the OE [42]. This suggests that bFGF and IGF-1 released from CGF act on basal cells via receptors in the OE and promote the induction of neurogenesis.

Topical injection of PRP into the olfactory cleft has recently been used in patients with olfactory dysfunction after COVID-19 and has been reported to improve the sense of smell [18–20]. Administration of PRP requires a submucosal injection into the olfactory cleft. Lechien et al. found no serious adverse events with its administration; however, patients complained of transient epistaxis (36%) and severe pain (19%) [19]. CGFs are a next-generation PRP prepared using a special centrifugation method. Preparation of CGFs is easier and faster than that of PRP, and CGFs are generated entirely from one’s own blood without anticoagulants. Similar to PRP, CGFs contain several growth factors including bFGF and IGF-1 that are released over time [38]

PRP is in the liquid form, whereas CGFs are in the gel form. Therefore, CGFs can be placed in the intact olfactory fissure without causing bleeding or pain. In basic research, methods of supplying growth factors to the OE have included intranasal drip infusion of bFGF [12] and administration using gelatin hydrogel [43], which is a drug delivery system that involves embedding a medicine. In particular, the administration of gelatin hydrogels is more effective than intranasal drip infusion in promoting olfactory epithelial regeneration [13, 44]; however, gelatin hydrogels have not been clinically applied to patients with olfactory dysfunction. CGFs release growth factors over 14 days in the absence of a gelatin hydrogel [38]. Therefore, CGFs may be more suitable than PRP or bFGF for the treatment of olfactory dysfunction. CGF has been clinically applied and shown to be effective [45]. Zhao et al. used CGF for mucosal defects of the nasal septum and reported efficacy and no side effects [46]. CGF appears to be clinically safe for use in nasal administration; however, its use requires safety confirmation because adverse events were not studied in this study.

In this study, there was no significant difference in the avoidance rates between the CGF and saline groups on day 28. The reason for the negative results of the avoidance test is probably because the olfactory epithelial thickness and the number of OMP-positive cells in the CGF group did not reach normal levels on day 28. However, Noda et al. observed up to 42 days of induced olfactory dysfunction in their models and found that the OE continued to regenerate [47]. In this study, there may be a possibility to improve olfactory behavior by extending the observation period.

Additionally, the OB was not evaluated in the present study. To assess the binding of ORNs in the OE to olfactory glomeruli, it is necessary to examine the OB volume and expressions of NGF and BDNF in the OB [10, 11]. Therefore, evaluation of the OB should also be performed.

## Conclusions

We evaluated the effects of intranasal CGF administration in a rat model of olfactory dysfunction. Histological examination showed that the CGF group showed improved olfactory epithelial thickness and OMP-positive cell numbers compared with that in the saline group.

## Supporting information

S1 Table Dataset of the avoidance rate. (PDF)

S2 Table Dataset of individual points and dataset for analysis of the OE thickness. (PDF)

S3 Table Dataset of individual points and dataset for analysis of OMP positive cells. (PDF)
