# Supplementary material for: Effect of intranasal administration of concentrated growth factors on regeneration of the olfactory epithelium in an olfactory dysfunction-induced rat model
Source: PLoS One. 2024 Feb 28;19(2):e0298640. doi: 10.1371/journal.pone.0298640 (PMC10901354; doi:10.1371/journal.pone.0298640)
Supplement: S1 Table — (PDF) [file pone.0298640.s001.pdf]

	Day-7		Day 0		Day 7		Day 14		Day 21		Day 28	
CGF-1	142<br>16	0.898734	86<br>12	0.877551	74<br>83	0.471338	92<br>60	0.605263	70<br>86	0.448718	90<br>62	0.592105
CGF-2	141<br>37	0.792135	51<br>54	0.485714	86<br>86	0.5	142<br>67	0.679426	77<br>102	0.430168	82<br>98	0.455556
CGF-3	165<br>68	0.708155	76<br>92	0.452381	128<br>91	0.584475	84<br>130	0.392523	185<br>86	0.682657	110<br>112	0.495495
CGF-4	136<br>31	0.814371	28<br>40	0.411765	117<br>76	0.606218	72<br>92	0.439024	130<br>66	0.663265	136<br>24	0.85
CGF-5	144<br>39	0.786885	80<br>35	0.695652	94<br>66	0.5875	108<br>57	0.654545	95<br>70	0.575758	88<br>86	0.505747
CGF-6	140<br>17	0.89172	22<br>20	0.52381	112<br>47	0.704403	94<br>54	0.635135	84<br>60	0.583333	78<br>76	0.506494
CGF-7	112<br>42	0.727273	50<br>30	0.625	102<br>32	0.761194	84<br>58	0.591549	104<br>35	0.748201	90<br>50	0.642857

	Day-7	Day 0	Day 7	Day 14	Day 21	Day 28
AVERAGE	0.802753	0.581696	0.602161	0.571067	0.5903	0.578322
STDEV.S	0.073341	0.163582	0.103151	0.110843	0.118838	0.135722
MEDIAN	0.792135	0.52381	0.5875	0.605263	0.583333	0.506494
MAX	0.898734	0.877551	0.761194	0.679426	0.748201	0.85
MIN	0.708155	0.411765	0.471338	0.392523	0.430168	0.455556

	Day-	
animal	intake of normal water<br>intake of vanillin water	avoidance rate

	Day-7		Day 0		Day 7		Day 14		Day 21		Day 28	
Saline-1	162<br>34	0.826531	68<br>50	0.576271	74<br>86	0.4625	102<br>103	0.497561	90<br>80	0.529412	97<br>94	0.507853
Saline-2	152<br>14	0.915663	78<br>12	0.866667	62<br>58	0.516667	112<br>36	0.756757	67<br>55	0.54918	113<br>22	0.837037
Saline-3	156<br>25	0.861878	40<br>25	0.615385	84<br>50	0.626866	96<br>88	0.521739	95<br>55	0.633333	80<br>85	0.484848
Saline-4	130<br>20	0.866667	48<br>12	0.8	80<br>70	0.533333	92<br>58	0.613333	100<br>42	0.704225	75<br>56	0.572519
Saline-5	126<br>40	0.759036	43<br>42	0.505882	88<br>68	0.564103	108<br>57	0.654545	95<br>56	0.629139	58<br>77	0.42963
Saline-6	157<br>46	0.773399	48<br>37	0.564706	155<br>81	0.65678	162<br>106	0.604478	170<br>90	0.653846	150<br>52	0.742574
Saline-7	120<br>50	0.705882	18<br>12	0.6	126<br>76	0.623762	148<br>50	0.747475	122<br>40	0.753086	130<br>32	0.802469

AVERAGE	0.815579	0.646987	0.569144	0.627984	0.636032	0.625276
STDEV.S	0.072915	0.133267	0.070035	0.100525	0.079199	0.165614
MEDIAN	0.826531	0.6	0.564103	0.613333	0.633333	0.572519
MAX	0.915663	0.866667	0.65678	0.756757	0.753086	0.837037
MIN	0.705882	0.505882	0.4625	0.497561	0.529412	0.42963

animal	Day-	
	intake of normal water<br>intake of vanillin water	avoidance rate
