# Supplementary material for: Effect of intranasal administration of concentrated growth factors on regeneration of the olfactory epithelium in an olfactory dysfunction-induced rat model
Source: PLoS One. 2024 Feb 28;19(2):e0298640. doi: 10.1371/journal.pone.0298640 (PMC10901354; doi:10.1371/journal.pone.0298640)
Supplement: S2 Table — (PDF) [file pone.0298640.s002.pdf]

Dataset of individual points of the OE thickness

		(μm)			(μm)			(μm)
normal-1	i	61.1	CGF-1	i	33.6	Saline-1	i	39.9
	ii	57.6		ii	41.1		ii	23.8
	iii	60.8		iii	37.9		iii	40.4
	ave.	59.83333		ave.	37.53333		ave.	34.7
normal-2	i	56.5	CGF-2	i	38.3	Saline-2	i	45.4
	ii	47.6		ii	42.9		ii	35.2
	iii	48.5		iii	28.9		iii	28.8
	ave.	50.86667		ave.	36.7		ave.	36.46667
normal-3	i	50.1	CGF-3	i	36.1	Saline-3	i	31.6
	ii	48.9		ii	35.9		ii	29.5
	iii	60.2		iii	32.9		iii	35
	ave.	53.06667		ave.	34.96667		ave.	32.03333
normal-4	i	53.9	CGF-4	i	40.9	Saline-4	i	40.4
	ii	56.5		ii	36.3		ii	35.2
	iii	45.5		iii	40.6		iii	35.1
	ave.	51.96667		ave.	39.26667		ave.	36.9
normal-5	i	61.1	CGF-5	i	49.2	Saline-5	i	26.6
	ii	48.9		ii	36.7		ii	31.3
	iii	52.9		iii	36		iii	31.8
	ave.	54.3		ave.	40.63333		ave.	29.9
			CGF-6	i	43.1	Saline-6	i	20.1
				ii	33.6		ii	22.8
				iii	33.9		iii	30.2
				ave.	36.86667		ave.	24.36667
			CGF-7	i	52.4	Saline-7	i	31.3
				ii	38.5		ii	38.1
				iii	42.6		iii	37.2
				ave.	44.5		ave.	35.53333

Dataset for analysis of the OE thickness

	Normal		CGF		Saline	
	1	59.83333	1	37.53333	1	34.7
	2	50.86667	2	36.7	2	36.46667
	3	53.06667	3	34.96667	3	32.03333
	4	51.96667	4	39.26667	4	36.9
	5	54.3	5	40.63333	5	29.9
			6	36.86667	6	24.36667
			7	44.5	7	35.53333
MEDIAN		53.06667		37.53333		32.03333
MAX		59.83333		44.5		36.9
MIN		50.86667		34.96667		24.36667
First quartile		51.96667		40.63333		36.46667
Third quartile		54.3		36.7		29.9
AVERAGE		54.08333		38.6381		32.84286
SD		3.357888		3.171016		4.498918
