# Supplementary material for: Effect of intranasal administration of concentrated growth factors on regeneration of the olfactory epithelium in an olfactory dysfunction-induced rat model
Source: PLoS One. 2024 Feb 28;19(2):e0298640. doi: 10.1371/journal.pone.0298640 (PMC10901354; doi:10.1371/journal.pone.0298640)
Supplement: S3 Table — (PDF) [file pone.0298640.s003.pdf]

Dataset of individual points of OMP positive cells

		$(/10^4 \mu m^2)$			$(/10^4 \mu m^2)$			$(/10^4 \mu m^2)$
normal-1	i	58	CGF-1	i	34	Saline-1	i	36
	ii	67		ii	31		ii	36
	iii	71		iii	31		iii	26
	ave.	63.33333		ave.	32		ave.	32.66667
normal-2	i	58	CGF-2	i	39	Saline-2	i	30
	ii	65		ii	36		ii	45
	iii	79		iii	32		iii	37
	ave.	67.33333		ave.	35.66667		ave.	37.33333
normal-3	i	48	CGF-3	i	49	Saline-3	i	30
	ii	61		ii	37		ii	25
	iii	62		iii	46		iii	28
	ave.	57		ave.	44		ave.	27.66667
normal-4	i	48	CGF-4	i	41	Saline-4	i	34
	ii	48		ii	39		ii	27
	iii	32		iii	35		iii	41
	ave.	42.66667		ave.	38.33333		ave.	34
normal-5	i	51	CGF-5	i	49	Saline-5	i	28
	ii	57		ii	60		ii	23
	iii	51		iii	56		iii	35
	ave.	53		ave.	55		ave.	28.66667
			CGF-6	i	44	Saline-6	i	27
				ii	41		ii	27
				iii	39		iii	29
				ave.	41.33333		ave.	27.66667
			CGF-7	i	39	Saline-7	i	28
				ii	50		ii	32
				iii	51		iii	26
				ave.	46.66667		ave.	28.66667

Dataset for analysis of OMP positive cells

	Normal		CGF		Saline	
	1	63.33333	1	32	1	32.66667
	2	67.33333	2	35.66667	2	37.33333
	3	57	3	44	3	27.66667
	4	42.66667	4	38.33333	4	34
	5	53	5	55	5	28.66667
			6	41.33333	6	27.66667
			7	46.66667	7	28.66667
MEDIAN		57		41.33333		28.66667
MAX		67.33333		55		37.33333
MIN		42.66667		32		27.66667
First quartile		53		35.66667		28.66667
Third quartile		63.33333		46.66667		34
AVERAGE		56.89667		40.29333		30.95238
SD		9.910876		9.772717		3.693512
